# *CalQuo*: automated, simultaneous single-cell and population-level quantification of global intracellular Ca^2+^ responses

**DOI:** 10.1038/srep16487

**Published:** 2015-11-13

**Authors:** Marco Fritzsche, Ricardo A. Fernandes, Huw Colin-York, Ana M. Santos, Steven F. Lee, B. Christoffer Lagerholm, Simon J. Davis, Christian Eggeling

**Affiliations:** 1 MRC Human Immunology Unit, Weatherall Institute of Molecular Medicine, University of Oxford, OX3 9DS Oxford, United Kingdom; 2 Wolfson Imaging Centre Oxford, Weatherall Institute of Molecular Medicine, University of Oxford, OX3 9DS Oxford, United Kingdom; 3 Department of Chemistry, University of Cambridge, CB2 1EW Cambridge, United Kingdom

## Abstract

Detecting intracellular calcium signaling with fluorescent calcium indicator dyes is often coupled with microscopy techniques to follow the activation state of non-excitable cells, including lymphocytes. However, the analysis of global intracellular calcium responses both at the single-cell level and in large ensembles simultaneously has yet to be automated. Here, we present a new software package, *CalQuo (*Calcium Quantification), which allows the automated analysis and simultaneous monitoring of global fluorescent calcium reporter-based signaling responses in up to 1000 single cells per experiment, at temporal resolutions of sub-seconds to seconds. *CalQuo* quantifies the number and fraction of responding cells, the temporal dependence of calcium signaling and provides global and individual calcium-reporter fluorescence intensity profiles. We demonstrate the utility of the new method by comparing the calcium-based signaling responses of genetically manipulated human lymphocytic cell lines.

Cellular calcium signaling is involved in most aspects of the cell’s life cycle^1^,^2^,^3^. Signaling occurs when the cell is stimulated to release calcium ions (Ca^2+^) from intracellular compartments and/or when Ca^2+^ enters the cell through calcium permeable channels^4^,^5^. Examples range from strongly localized Ca^2+^ sparks or spikes accompanying contraction in cardiac muscle cells or synaptic signaling in neuronal cells, to more global responses in non-excitable cells such as lymphocytes. In view of the large extracellular Ca^2+^ levels (>1 mM), cells must invest significant resources to maintain and to drive changes in the relatively low cytoplasmic Ca^2+^ levels (10–100 nM). This is especially true when calcium channels in the endoplasmic reticulum (ER) or the plasma membrane open during signaling, and the cytoplasmic Ca^2+^ concentration increases 10–100 fold^5^. In the case of non-excitable cells, triggering of cell surface protein receptors often leads to a sudden increase in cytoplasmic calcium levels^5^. Triggering of G-protein-coupled receptors, tyrosine kinase-coupled receptors, such as growth factor receptors and, in the case of leukocytes, non-catalytic tyrosine-phosphorylated receptors, such as B- and T-cell receptors activate phospholipase C (PLC) which hydrolyzes the membrane phospholipid PIP2 to form inositol 1,4,5-triphospate (IP3). IP3 then diffuses to the ER where it binds to the IP3 calcium channel triggering the release of Ca^2+^ 2,4,6.

Despite the identification and extensive characterization of the signaling pathways initiated by receptor triggering, and the central importance calcium plays in these events, the automated real-time quantification of calcium dynamics is generally not straightforward. Calcium responses are rapid and usually transient, follow many different pathways, and can vary significantly from cell to cell. Such dynamic and variable responses place considerable demands on the quantitative analysis of stimulus-elicited changes in cytoplasmic Ca^2+^ levels. While various automated analysis methods have been proposed to quantify Ca^2+^ levels or to resolve strongly localized Ca^2+^ sparks or spikes in *e.g.* the cytoplasm of cardiac muscle cells or neurons^7^,^8^,^9^,^10^,^11^,^12^, similar approaches have not yet been applied to study IP3-mediated Ca^2+^ responses triggered by cell surface receptors expressed by non-excitable cells, such as leukocytes. Here, Ca^2+^ “responses” are defined as global intracellular Ca^2+^ level increases that are transient on the sub-second to second time scale. Preferably, it needs to be possible to allow different routes of stimulation (*e.g. via* functionalized surfaces, cell-cell contact or controlled addition of small molecules) and to detect and distinguish calcium responses by individual cells within cell populations, at several micrometer spatial resolution and sub-second temporal resolution non-invasively. Common live-cell approaches rely on the use of fluorescence reporters that change emission properties upon Ca^2+^ binding^13^,^14^. Typically, quantitation of Ca^2+^ responses involves observing a small number of cells (<100) under a microscope, or in a flow cytometer or micro-plate reader. These approaches are limited in scope and struggle especially to quantitate individual cell behavior within large ensembles of cells. Additional issues that often arise include the detection sensitivity and/or sampling noise^4^,^15^. Recently, quantification of global Ca^2+^ responses of individual cells within an ensemble has been achieved by using fluorescence microscopy with single-cell resolution in combination with different software packages^16^,^17^. However, automation has yet to be fully incorporated into these analyses, and instead time-consuming, user-dependent, manual procedures had to be used, supported occasionally by commercial software packages of limited capability such as Microsoft Excel^13^. To overcome these limitations and to facilitate the automated quantification of global intracellular Ca^2+^ responses we developed a bespoke MATLAB-based software for Calcium Quantification (*CalQuo*). *CalQuo* allows automated, real-time determination of global calcium responses in hundreds of individual cells simultaneously, using conventional fluorescence microscopes, in this case a spinning-disk confocal scanning microscope.

We exemplified our approach by examining the calcium responses of T-cells settling on functionalized microscope cover glass. The release of intracellular Ca^2+^ is one of the earliest steps in T-cell activation and a well-established maker for early T-cell receptor (TCR) triggering^13^,^14^. Because early T-cell activation events modulate the course of the adaptive immune response, the quantification of cytoplasmic Ca^2+^ has been an important tool in the study of these processes^13^. We measured changes in cytoplasmic Ca^2+^ concentration using the fluorescein-based tetracarboxylate chelator Fluo-4, a Ca^2+^ fluorescent reporter, which upon binding to divalent cations increases its fluorescence emission, in the case of calcium, up to 100 fold^18^,^19^. We graphically visualize the Fluo-4 calcium transients corresponding to the calcium responses by monitoring the fluorescence intensity profiles of individual T-cells. Equipped with a low 10 x magnification and high numerical-aperture (NA = 0.45) objective and a 50 μm sized pinhole, the spinning disc microscope allows high-speed imaging of a large field of view (900 × 900 μm^2^) with hundreds of cells at single-cell spatial resolution simultaneously. In the present experiments the focal plane was placed at the glass surface and we recorded 870 images at a frame rate of 2 Hz (Supplementary Materials). In addition to image acquisition speed, the spinning disk microscope has the advantage of minimizing detection of out-of-focus fluorescence signals, allowing cells at and above the glass surface to be distinguished.

*CalQuo* uses feature recognition and distance regularized level set evolution (DRLS) algorithms^20^,^21^,^22^ to segment the raw-data image stacks and uses the calcium-dependent fluorescence signal to detect both calcium levels and cell features (see Supplementary Materials). Specifically, *CalQuo* detects cell motion, including the moment cells interact with, or “land” on the surface. To visualize the calcium responses following these landing events, we generated maximum projections of the time-lapse images with the detected fluorescence intensity color-coded from blue (low) to red (high calcium), as shown in Fig. 1a. The images reveal the motion of individual cells over time in suspension, allowing landing cells to be identified by an immediate cessation of movement, with the subsequent calcium response represented by a sudden increase in the fluorescence signal.

Cells that reached the surface in the first 300 s and were within the field of view (and therefore not closer than one cell diameter from the outer edges) were identified and selected by *CalQuo* for further analysis. We specifically excluded events where cell boundaries overlap at the same position. Such events were <2% due to choosing a suitable cell density. In this way, *CalQuo* was able to record the calcium responses for virtually every cell in the field, *i.e.* the fluorescence intensities *I*(*t*) over time *t* (Fig. 1b and Fig. S1). For our analysis, we interpolated and normalized the individual raw data curves using the Savitzky-Golay and normalised-data interpolation (see Supplementary Materials), defining the relative fluorescent intensity *R*(*t*) = *I*(*t*)/*I*_*max*_, where *I*_*max*_ is the maximum intensity value of the response curve (Fig. 1b). To follow the calcium responses of individual cells, *R*(*t*) was averaged across each cell. In the next step, *CalQuo* identified the landing time and the beginning of the calcium response (described in more detail in Supplementary Materials). Both events led to an increase in fluorescence signal and therefore became prominent as two separate peaks in the derivative d*R*(*t*)/d*t* of the response function (Fig. 1c). The increase in fluorescence signal due to landing was relatively small and was the result of the weakly fluorescent cells reaching the focal plane of the objective located at the glass surface. In contrast, cell signaling resulted in the strong calcium-dependent increase of the fluorescence emission of Fluo-4. Signaling cells were thus identified by a characteristic sharp increase in fluorescence (after landing) followed by a slow decrease, while non-signaling cells exhibited little or no change in fluorescence signal. To allow direct comparisons of the responses of individual cells, the time axes of all the response curves *R*(*t*) were aligned, with time *t* = 0 set to 2 s after the landing event (Fig. 1c). *CalQuo* therefore allowed us to (1) identify the number and fraction of triggered cells, *i.e.* those showing a >500% increase above background (arbitrarily set by the user) and subsequent decrease in *R*(*t*); and (2) determine a response profile *R*(*t*) averaged over all activated cells, with error bars reflecting the cell-to-cell variation in responses (Fig. 1c).

To demonstrate the capabilities of *CalQuo*, we characterized the calcium responses of Jurkat T-cells and a calcium signaling-deficient derivative, J.Cam1.6, which is a T-cell line deficient in the Lck kinase due to partial deletion of the Lck gene^23^,^24^,^25^. J.Cam1.6 cells are unresponsive to T-cell receptor (TCR) activation and therefore exhibit impaired calcium responses upon TCR-ligand binding^23^,^25^. Both Jurkats and J.Cam1.6 cells were labeled with the Fluo-4 dye and allowed to interact with a glass surface coated with stimulating anti-CD3ε (OKT3) and anti-CD28 (CD28.2) antibodies at 37 °C for ~400 s^25^,^26^. *CalQuo* detected clear differences in the fractions of responding Jurkats and J.Cam1.6 cells (Fig. 1a, left panels): whereas 60% of Jurkat T-cells exhibited an increase in Fluo-4 intensity upon surface contact and thus antibody binding (Fig. 2 and Movie S1), only 20% of the J.Cam1.6 cells showed a similar response (Fig. 2 and Movie S2). Moreover, the average response curve *R*(*t*) obtained for the Jurkat T-cells exhibited the characteristic sharp increase in fluorescence followed by a slow decrease, while that of J.Cam1.6 cells was essentially flat (Fig. 2a). Upon transfection of wild-type Lck, 40% of signaling competent J.Cam1.6 cells showed the characteristic calcium response following TCR triggering (J.Cam1.6 wthLCK, Fig. 1a right top panel, Fig. 2 and Movie S3)^23^. Conversely, Lck-expressing J.Cam1.6 cells where the expression of the TCRβ chain, and therefore of the TCR complex, was reduced using shRNA exhibited severely compromised calcium responses upon stimulation (J.Cam1.6 TCRk_d_LCK, Fig1a right lower panel, 10% responses, Fig. 2 and Movie S4).

By determining the exact times of stimulation (*i.e.* landing) and signaling (i.e. calcium release) using *R*(*t*), *CalQuo* allowed us to measure the time interval between these two events, *T* = *t*_*triggering*_ – *t*_*landing*_, which is another parameter characterizing the calcium signaling response, and is particularly useful for characterizing the agonist potential of putative receptor ligands. In Fig. 3a a histogram showing *T* for the four sets of T-cells responding to stimulating conditions is shown, and Fig. 3b depicts boxplots of the triggering intervals. Overall, Jurkat T-cells showed the highest triggering fraction and the fastest TCR triggering responses (Table 1), followed by signaling-restored J.Cam1.6 cells (J.Cam1.6 wthLCK). As expected both J.Cam1.6 and J.Cam1.6 cells lacking TCRβ chains (J.Cam1.6 TCRk_d_LCK) exhibited significant delays in TCR triggering (Table 1).

We also tested the impact of time resolution on the analysis. We analyzed our data mimicking different time resolutions from 0.5 s to 60 s (Fig. 1d). The fraction of positive Ca^2+^ responses was significantly lower at the slower acquisition times, with >1 s fast recordings as used elsewhere^13^,^17^ partially or even fully failing to detect responses (Fig. 1e).

While *CalQuo* allowed us to quantify with high statistical accuracy fractions of Ca^2+^ responding cells within a cell ensemble, the investigated responses were averaged over the whole cell. *CalQuo* is therefore ideally suited to characterize receptor triggering in non-excitable immune cells. In its current formulation, *CalQuo* is unable to resolve Ca^2+^ “sparks”, usually occurring locally within a cell (sub-micrometer) within milliseconds, or quantify absolute Ca^2+^ concentrations. However, *CalQuo* allowed us to determine the contribution of differences in Fluo-4 dye loading and/or background levels to the global responses under analysis. To this end, we determined absolute (*i.e.* non-normalized) fluorescence intensity levels of the basal and maximum signal, *I_0_* and d*I* = *Imax* – *I_0_*, respectively, at single-cell level. Values of d*I/I_0_* did not change in the course of our study, *i.e.* Jurkat, J.Cam 1.6, J.Cam1.6 wthLCK and J.Cam1.6 TCRk_d_LCK (Fig. S2), indicating that our readout was robust against dye loading or background levels. Furthermore, the determined response curves were largely unaffected by collecting 2500 to 20 camera pixels per cell (Fig. S3), suggesting that, in principle, *CalQuo* could be used with a 5 x magnification objective lens. Indeed, the fraction of triggered cells was unaffected by moving from the 10 x objective lens to a 40 x or even 60 x objective lens on our spinning-disc microscope (Fig. S4).

In conclusion, we have developed a robust method for the automated quantification of global calcium responses of individual cells within relatively large populations. Combining feature detection and the novel DRLS algorithms with our response curve filtering algorithms offers unprecedented accuracy in segmenting the raw images for cell feature detection and reliable quantification of the calcium responses. The use of *CalQuo* in combination with fast spinning-disc microscopy and fluorescent calcium reporters allowed the quantification of the number (or fraction) of calcium-releasing cells, including the time-dependent global calcium responses, and the interval between stimulation and calcium release. At present the software requires a minimal level of MATLAB-based operating knowledge.

*CalQuo* is well suited to studying other cell signaling processes, such as the release of proteins or other molecules (*e.g.* toxins), if these molecules can be detected using fluorescent labels. We expect *CalQuo* to contribute to a more facile and thus better understanding of the molecular basis of global signaling in eukaryotic cells.

## Additional Information

**How to cite this article**: Fritzsche, M. *et al.* *CalQuo*: automated, simultaneous single-cell and population-level quantification of global intracellular Ca^2+^ responses. *Sci. Rep.* **5**, 16487; doi: 10.1038/srep16487 (2015).

## Supplementary Material

Supplementary Information

## Figures and Tables

**Figure 1:**
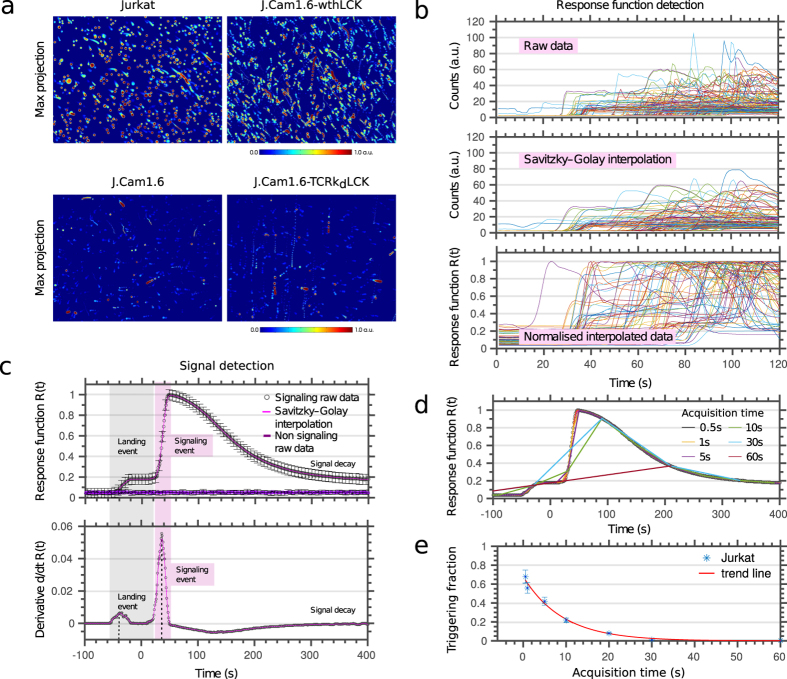
Observing global calcium release in T-cells using *CalQuo*. (**a**) Maximum projections of fluorescence intensity over time from Fluo-4 loaded Jurkat (upper left), J.Cam1.6 (lower left), J.Cam1.6-wthLCK with restored signaling ability (upper right), and J.Cam1.6-TCRbk_d_-Lck with reduced TCRβ chain (lower right) when landing on the activating antibody-coated microscope cover glass, as measured by a spinning disc confocal fluorescence microscope, whose sampling plane was put on the cover glass. Low to warm colors represent low to high fluorescence intensities. Moving and triggering T-cells can be identified by a red dot with a blue tail. (**b**) Raw response data *R*(*t*) from different individual cells demonstrating the different analysis step: raw data (upper panel), data following Savitzky–Golay interpolation (middle panel) and normalised interpolated data (lower panel). (**c)** Representative intensity profiles *R*(*t*) (upper panel) and their derivatives d*R*(*t*)/d*t* (lower panel); average over 200-650 individual T-cells (black circles: raw data, mangenta line: Savitzky–Golay interpolation for signaling T-cells, purple line: data of non-signaling cells). *CalQuo* determines the characteristic times for the landing (grey shaded area) and signaling event (magenta shaded area) from the characteristic peaks in d*R*(*t*)/d*t*. Error bars representing s.d.m. (**d**) Representative response functions *R*(*t*) of a calcium releasing cell analyzed for different time resolutions 0.5 s–60 s, as indicated. The ability to identify calcium response decreases with decreasing time resolution, as revealed by (**e**) the fraction of signaling, *i.e.* calcium-releasing cells determined from the same data set for different time resolutions as shown in (**e**).

**Figure 2:**
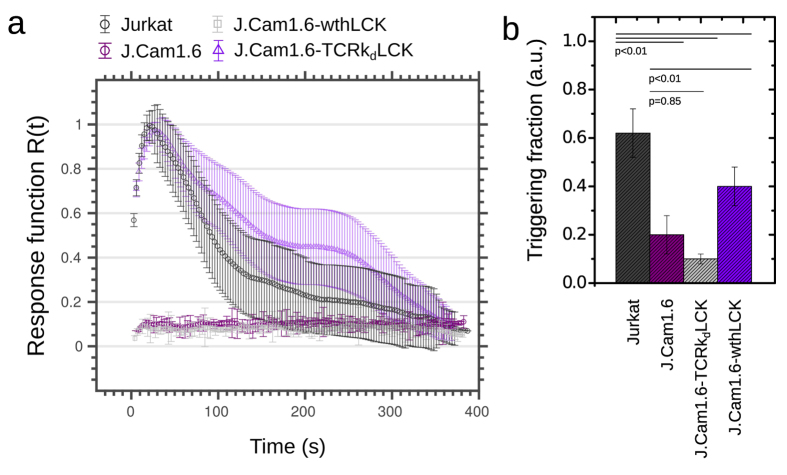
*CalQuo* output for the different types of T-cells. Average response functions *R*(*t*) (**a**) and fraction of signaling, *i.e.* calcium-releasing cells (**b**). Error bars as s.d.m. over 200–650 cells (see Table 1).

**Figure 3:**
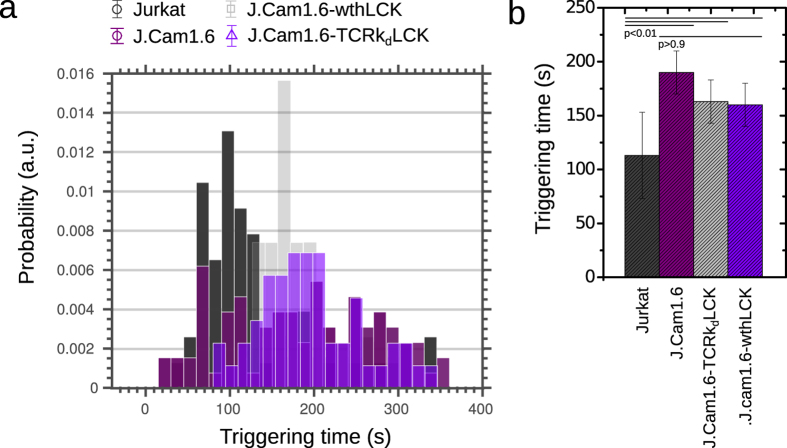
*CalQuo* output for the different types of T-cells: Triggering time *T* (*i.e.* time between landing and signaling) for the fraction of signaling cells; histogram (**a**) and corresponding boxplots (**b**). Error bars as s.d.m. over 200–650 cells (see Table 1).

**Table 1:** Average and s.d.m. of the fraction of calcium responding cells and the triggering times *T* for the different types of T-cells (*N* = number of cells investigated).

Experiment	Triggeringfraction	Triggeringtime	N
Jurkat	0.6 ± 0.1	113 ± 40	366
J.Caml.6	0.2 ± 0.1	191 ± 30	629
J.Caml.6-wthLCK	0.4 ± 0.1	158 ± 20	302
J.Caml.6-TCRk_*d*_LCK	0.1 ± 0.01	163 ± 20	213
