# Supplementary material for: CalQuo: automated, simultaneous single-cell and population-level quantification of global intracellular Ca2+ responses
Source: Sci Rep. 2015 Nov 13;5:16487. doi: 10.1038/srep16487 (PMC4643230; doi:10.1038/srep16487)
Supplement: Supplementary Information [file srep16487-s1.pdf]

SUPPLEMENTARY MATERIALS

CalQuo: automated, simultaneous single-cell and population-level quantification of intracellular Ca^{2+} responses

Marco Fritzsche[1§], Ricardo A. Fernandes[1,4§], Huw Colin-York[1], Ana M. Santos[1], Steven F. Lee[3], Christoffer B. Lagerholm[2], Simon J. Davis[1], and Christian Eggeling[1,2]

[1]MRC Human Immunology Unit and [2]Wolfson Imaging Centre Oxford, Weatherall Institute of Molecular Medicine, University of Oxford, OX3 9DS Oxford, United Kingdom.

[3]Department of Chemistry, University of Cambridge, CB2 1EW Cambridge, United Kingdom.

[4]Current address: Department of Molecular & Cellular Physiology, Stanford University School of Medicine, Stanford, CA 94305

[§] Correspondence: marco.fritzsche@rdm.ox.ac.uk and ricardfe@stanford.edu

MATERIALS AND METHODS

Cell culture. Jurkat and JCaM 1.6 T-cells were cultured in sterile RPMI 1640 (Sigma Aldrich) supplemented with 10% FCS (PAA), 2mM L-Glutamine (Sigma Aldrich), 1mM Sodium Pyruvate (Sigma Aldrich), 10mM HEPES (Sigma Aldrich), and 1% Penicillin-Streptomycin-Neomycin solution (Sigma Aldrich). Cells were maintained at 37°C and 5% CO2 during culturing, and handling was performed in HEPA-filtered microbiological safety cabinets. Cells were kept at density between 5-9 x 10^5/ml.

Lentiviral Infections. Lentiviral infections were used to generate stably transfected T-cell lines expressing the desired genes of interest. HEK 293T cells, grown in DMEM supplemented with 10% FCS, 1% HEPES buffer, 1% sodium pyruvate and 1% antibiotics, were plated in 6-well plates, 6x10^5 cells/well (all reagents from Sigma Aldrich, Dorset, UK). After 24 hours HEK 293T cells were transfected with equal amounts of pHR plasmid (including the gene of interest), p8.91 and pMDG using Genejuice (Novagen Merck, Hull, UK) as per manufacturer's recommendations (1). 48 hours post-transfection the supernatant from two 6-well plates was added to 1x10^6 T cells.

Vector Constructs. The sequences of all genes were amplified using PCR from cDNA, IMAGE sequences or previously cloned vectors. For wild-type human Lck the stop codon was mutated for an appropriate restriction enzyme and the gene was inserted, in frame, at the N-Terminus of HALO® tag DNA sequence (Promega) using a modified pHR plasmid. For TCRβ knockdown the pHR plasmid was adapted for siRNA expression by changing the SFFV promoter sequence for a U6 promoter (Pol III promoter; kindly provided by Alain Townsend, WIMM, University of Oxford). A suitable shRNA target for TCRβ was found after searching for the following sequence: N2[GC]N[A]N6[UT]N2[ATUC]N5[A]N2 (2-7), on TCRβ mRNA using siRNA at Whitehead software (http://sirna.wi.mit.edu/). The final siRNA expression plasmid consists of a nucleotide sense sequence (GACTGGAGTTGCTCATTTA), followed by a loop (TTCAAGAGA), an antisense sequence (TAAATGAGCAACTCCAGTC) and a stop signal consisting of a stretch of five Ts. The appropriate shRNA was cloned into the lentiviral U6 promoter using BamHI and EcoRI restriction enzymes. All genes were sequenced to confirm reading frame and sequence integrity.

Activating-surfaces. Microscope coverslips were functionalised for T-cell activation by coating with αCD3ε and αCD28 antibodies. 25mm diameter coverslips (SLS) were coated with 1ml 50µg/ml donkey anti-mouse IgG antibody in coating buffer (50mM Na2CO3, 50mM NaHCO3,pH 9.6, filtered using a 0.22µm Millex®-GP syringe filter unit) at 4°C. Coverslips were washed with 3 x 1ml PBS and blocked for 3 h at room temperature using RPMI + 10% FCS. Coverslips were again washed with 3 x 1ml PBS and coated with 1ml mouse αCD3ε (Jackson Immunoresearch, clone: OKT3) and mouse αCD28 (eBioscience, clone: CD28.2) at 1µg/ml each plus OX7 IgG at 1µg/ml in HBS at 4°C overnight. Coverslips were washed a final time with 3 x 1ml HBS before use.

Calcium triggering protocol. All cells were labeled with 4µM Fluo-4 AM (F-14201; Invitrogen, Paisley UK) for 30min at room temperature with 2.5mM probenecid (P-36400; Invitrogen, Paisley UK) in RPMI (Sigma-Aldrich, UK) without supplements. Cells were then washed in HBS (51558; Sigma, UK) and medium changed to HBS containing 2.5mM probenecid before adding to antibody-coated slides or to SLBs. Cells were imaged using 10x objective and a NA=0.45 on a spinning disk confocal microscope (Carl Zeiss AG, Overkochen, Germany) under 488 nm laser excitation, with an exposure time of 350 ms and a time between frames of 500 ms for 840 frames.

Protein expression. Lck and TCRβ expression was quantified by FACS using a Beckman Coulter CyAn Analyser. For surface labelling of TCR complex an anti-CD3ε-alexa647 was used (purified from hybridoma supernatant, clone UCHT1 and labeled with antibody labeling kit from Molecular Probes, Invitrogen). For the intracellular staining of Lck an anti-Lck antibody (clone 73A5, Cell Signalling) was used. Lentiviral infected JCaM 1.6 T cells were fixed in 1% formaldehyde for 15 minutes at room temperature. Cells were then incubated with appropriate dilutions of the antibody. Primary antibody was detected using a donkey anti-rabbit-APC (Molecular Probes, Invitrogen) antibody. Jcam 1.6 cells expressing Lck-Halo were also stained with HaloTag® TMR ligand (Promega) as per manufacturer recommendation.

Calcium triggering analysis. CalQuo Software was used to detect cell landing events on protein-coated glass surfaces and record fluorescence intensities for each recorded frame. Sharp changes in fluorescence intensity above background levels (>3 fold) were indicative of calcium releases associated with TCR triggering in T-cells. Time lapse between "landing" and "triggering" events were also obtained directly from the CalQuo output.

CALQUO SOFTWARE

The CalQuo Software is written in the latest release of MATLAB version R2015a (Mathworks, UK) and was licensed with Isis Innovation (isis-innovation.com) permitting reuse within proprietary software provided all copies of the licensed software include a copy of the Isis License terms and the copyright notice.

SOFTWARE DESCRIPTION

The CalQuo Software package consists of two programs 'CalQuo_masterfile.m' and 'CalQuoAnalysis_masterfile.m'. 'CalQuo_masterfile.m' reads multiple 8bit stack-files in tif-format from a user-defined folder. The image stacks can comprise different pixel sizes and cell densities but need to have the same number of time frames. Subsequently, 'CalQuo_Analysis.m' further analyses the output data in the MATLAB workspace of 'CalQuo_masterfile.m'. Relevant parameters for software control and selection of appropriate signaling response functions are edited in 'CalQuo_parameters.m' present in the main folder. This parameter file is independently read by both programs 'CalQuo_masterfile.m' and 'CalQuoAnalysis_masterfile.m'. This way, the user can dynamically adjust and edit relevant parameters after the raw-data were read in and workspaces were calculated. It is therefore recommended to save the original workspaces after raw-data reading to then optimize analysis parameters without the need to pre-process the raw images again. The output of CalQuo is organized in file-structures and can be found in the MATLAB workspace saved as the structure QUANTDATA including the sub-structure STATISTICS and files. STATISTICS comprises the relevant parameters: number of triggering cells including the averaged response function, the time-points of signaling, and the decay times of the calcium signal. The parameters are given for the individual cells as well as an average over all cells (or multiple files) along with standard statistical parameters such as standard deviations. The corresponding values for the individual files can be found in the structure files.

'CalQuo_masterfile.m' uses feature recognition (FR, MATLAB function ‘findfeature2d’, see http://people.umass.edu/kilfoil/downloads.html) and the novel distance regularized level set evolution (DRLS) algorithms to segment the rawdata image stacks and detect the cell features such as the signaling response functions as detailed in [16-18]. The user can run the cell feature detection in two different modes: the first mode uses the feature edge for determination of the cell’s location and the second mode uses a rough estimation of the cell periphery, a more robust approach for cells drifting within the time-coarse of the experiment. In practice, the FR algorithm splits each raw-data image in the image stack into multiple sub-regions where it recognizes the cell features. Subsequently, the DRLS algorithm finds the precise location and edges of the cell features. The users can decide to exclude very bright and fluorescence saturated cells with the feature threshold parameter because the curves can alter the average response behavior. For example, one can set the ‘feature_thresh=0.98’ to deselect only the 2% brightest cells in the image stack. The feature recognition function requires the user to set three parameters that can be directly determined from the raw data. The first parameter is the typical ‘feature_size’ in pixels (i.e. the cell size), the second parameter is the approximate ‘subregion_size’ in arbitrary units, and the third parameter is the ‘feature_thresh’ in percentage intensity units.

CalQuo’s DRLS algorithm uses a dynamically shrinking polygon to find the feature edges and then calculate their intensity profiles in time. For this, the user is asked to define an approximate minimal and maximal size of the inner and outer edge of the feature cells with the two parameters ‘iter_inner ‘and ’iter_outer’.

SOFTWARE CONTROL

Optimal DRLS and FR parameters allow sufficient image segmentation and feature recognition but are limited in the selection of representative calcium response curves. The software does not include dynamic feature tracking and therefore relies on minimal movements of the cells once they have been detected. In the section ‘Parameters software control’ in the function 'CalQuo_parameters.m', the user is asked to define a name of the experiment and the frame-rate as well as the first and last frame number for the data analysis. The parameter ‘firstframenumber’ defines the frame at which the cell features are recognized and further analyzed by the FR and DRLS algorithms. Note, the parameter ‘firstframenumber’ is defined as the number of frames minus one while the second parameter ‘lastframenumber’ equals the total number of frames. If the parameter ‘lastframenumber’ is chosen to be smaller than the total number of frames, the remaining frames are excluded from the analysis.

CALIBRATION AND SELECTION OF CALCIUM RESPONSE FUNCTIONS

In the section ‘Parameters Profile control’ in the function 'CalQuo_parameters.m', the user is asked to set the five parameters alpha, beta, gamma, delta, and epsilon to sufficiently calibrate the selection automation of the software (Fig. S1). All parameters can be read off and dynamically adjusted response curves by running the 'CalQuoAnalysis_masterfile.m' multiple times until readout data such as the signaling fractions and response curves no longer improves. The parameter alpha defines the ratio of fluorescence intensities between the landing plateau and background signal in arbitrary units at early time-points prior landing. The parameter beta equals the approximate intensity value at the frame of calcium triggering in the units counts where the fluorescence peak originates. The parameter gamma defines the minimum ratio of fluorescence intensity of the maximum peak and the average landing intensity at long times in arbitrary units. This parameter can be deactivated by setting it to zero. The parameter delta defines the minimum fluorescence intensity ratio of the last and first ten frames in arbitrary units. The last parameter epsilon defines the minimum distance between landing and triggering time in the units’ seconds.

Running the CALQUO SOFTWARE

'CalQuo_masterfile.m' can be started by typing its name into the MATLAB command line and pressing enter. Subsequently, 'CalQuoAnalysis_masterfile.m' is run by typing its name into the MATLAB command line and pressing enter as well. All relevant output parameters are saved to the workspace as described above. The distribution of triggering times pops up in form of a histogram in counts as a function of time in seconds and the averaged response curves are presented in graph showing normalized intensities as a function of time in seconds.

SUPPLEMENTARY FIGURES

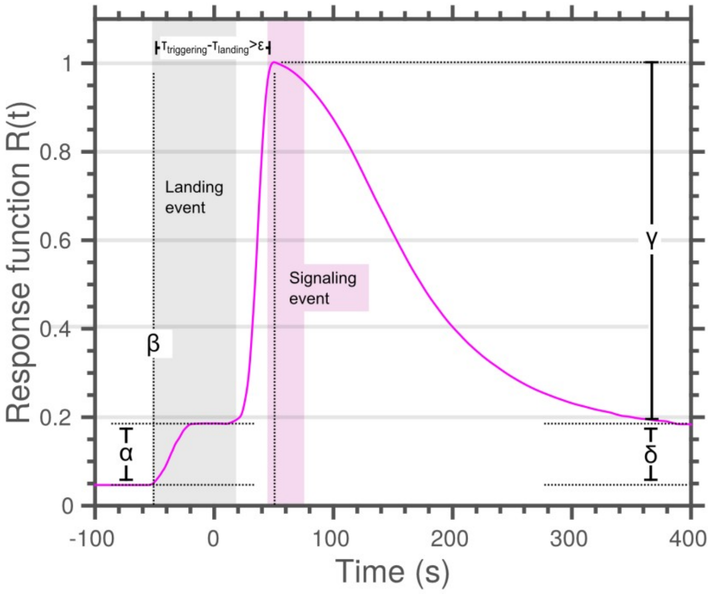

Fig. S1: Selection parameters for response curves R(t). The five parameters α, β, γ, δ, and ε allow the user to select the response functions.

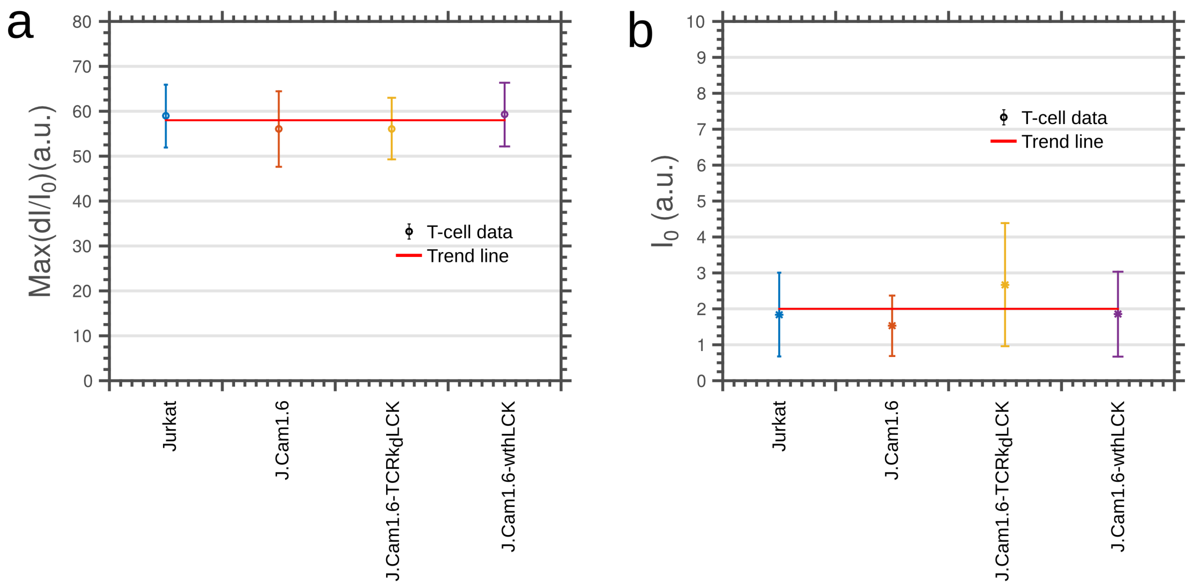

Fig. S2: Dye loading and background signal levels were similar across the different T-cell line conditions (as labeled). we determined for each cell absolute (i.e. un-normalized) fluorescence intensity levels of the basal and maximum signal, I_0 and $dI = I_{max} - I_0$, respectively. Both the peak (a) and the basal levels (b) of the response curves $dI(t)/I_0$ did not change for the different experimental conditions. Error bars = s.d.m. from averaging over all investigated cells (for total numbers of cells see Table 1).

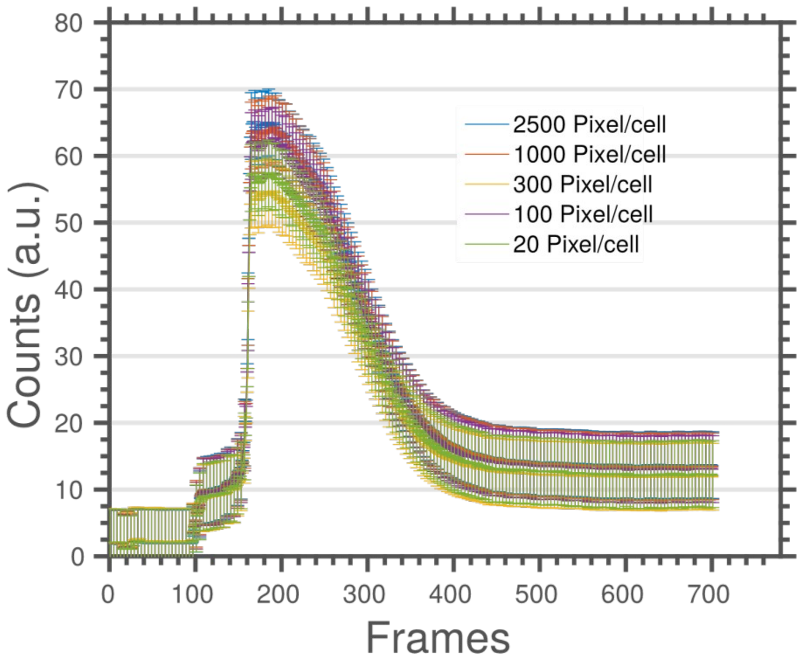

Fig. S3: Average over all response curves I(t) in Jurkat T-cells generated from the image data with increasing pixel size, resulting in less pixels per cell, as labeled. I(t) was hardly affected by the number of pixels per cell at our experimental setup (300 pixels per cell in our case with the 10x microscope objective). This result indicates that a magnification of down to 5x would have been sufficient to accurately detect the calcium responses. Error bars = s.d.m. resulting from averaging over N = 366 cells.

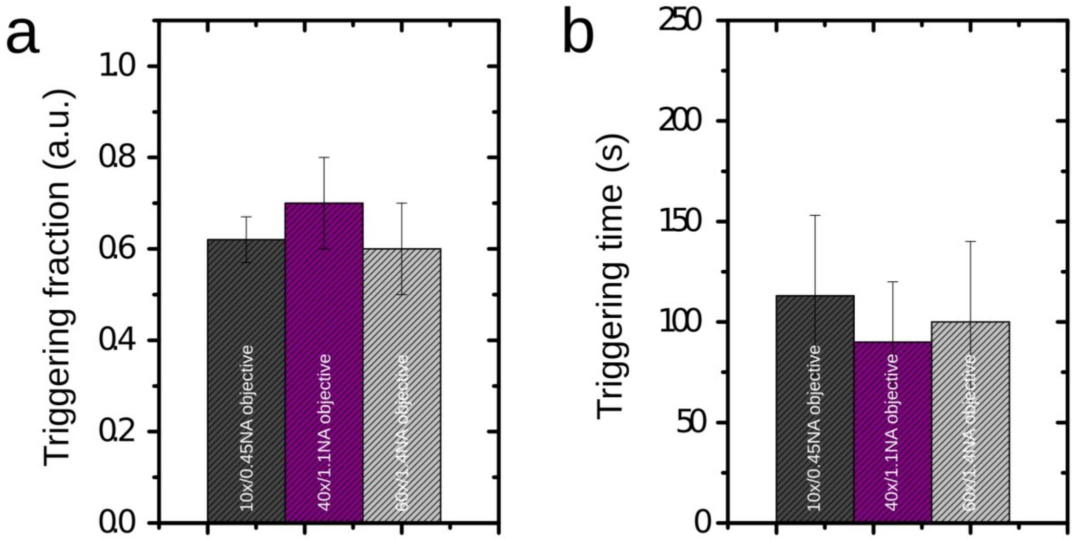

Fig. S4: The experimentally determined values (using *CalcQuo*) in fraction of triggered Jurkat T-cells (a) and triggering time T (i.e. time between landing and signaling) for the fraction of signaling Jurkat T-cells (b) were independent of the employed microscope objective (with different magnification and numerical aperture NA, as labeled; $p>0.94$ compared to 10x/0.45NA objective conditions). Error bars = s.d.m. over $N > 200$ cells.

SUPPORTING MOVIES

Supplementary Movie S1. Time-lapse images of Fluo4 fluorescence (maximum projection over 780 frames) detected for Jurkat T-cells when landing on microscope cover glass coated with αCD3ε and αCD28 antibodies. The cells were pipetted onto the culture medium of the imaging dish and then imaged. The majority of cells stopped movement and flashed brightly when touching the glass surface indicating calcium triggering. Scale bar: 100 µm. Total duration: ~6 min.

Supplementary Movie S2. Time-lapse images of Fluo4 fluorescence (maximum projection over 780 frames) detected for J.Cam1.6 T-cells when landing on microscope glass coated with αCD3ε and αCD28 antibodies. The cells were pipetted onto the culture medium of the imaging dish and then imaged. The majority of cells stopped movement but did not flash when touching the glass surface, indicating the absence of calcium release. Scale bar: 100 µm. Total duration: ~6 min.

Supplementary Movie S3. Time-lapse images of Fluo4 fluorescence (maximum projection over 780 frames) detected for J.Cam1.6-wthLCK T-cells when landing on microscope glass coated with αCD3ε and αCD28 antibodies. The cells were pipetted onto the culture medium of the imaging dish and then imaged. The majority of cells stopped movement and flashed when touching the glass surface, indicating calcium triggering. Scale bar: 100 µm. Total duration: ~6 min.

Supplementary Movie S4. Time-lapse images of Fluo4 fluorescence (maximum projection over 780 frames) detected for J.Cam1.6-TCRβk_d -Lck T-cells when landing on microscope cover glass coated with αCD3ε and αCD28 antibodies. The cells were pipetted onto the culture medium of the imaging dish and then imaged. The majority of cells stopped movement but did not flash when touching the glass surface, indicating the absence of calcium release. Scale bar 100 µm. Total duration: ~6 min.
